# Strategies to Minimize Virus Transmission During Anesthesia Procedures in COVID-19 Patients

**DOI:** 10.26502/acc.071

**Published:** 2024-10-23

**Authors:** Fihr Chaudhary, Devendra K Agrawal

**Affiliations:** Department of Translational Research, College of Osteopathic Medicine of the Pacific,Western University of Health Sciences, Pomona CA 91766, USA

**Keywords:** Aerosolization, Airway Management, Anesthesia, Cardiac Anesthesia, COVID-19, Healthcare Workers, Immunomodulation, Intubation, Mechanical Ventilation, Obstetric Anesthesia, Personal Protective Equipment, Rapid Sequence Induction, Regional Anesthesia, SARS-CoV-2, Virus Transmission

## Abstract

Anesthesiologists and the critical care team may be at increased risk of contracting severe acute respiratory syndrome coronavirus-2 (SARS-CoV-2, COVID-19) due to airway manipulations and intubations performed during anesthesia administration and management of patient undergoing surgery. SARS-CoV-2 infections have been reported among healthcare workers. The virus is transmitted by close personal contact and aerosols. During intubation and other procedures involving the airway, the anesthesiologist is especially susceptible to aerosols. We performed a systematic analysis of the published reports on potential effects of COVID-19 during surgery on the anesthesiologist and critical care team. and identified potential immunomodulatory effects of general anesthetics in the presence of COVID-19 infection in patients. The article also provides critical discussion on the current medical management of COVID-19 and highlights the evidence-based key points for a safer practice during anesthesia administration and surgeries both in children and adults, including obstetric procedures and how it could affect pregnant women receiving anesthesia. With regional anesthesia, airway manipulation is not necessary, and healthcare workers and other patients are less likely to contract the same infection.

## 1. Introduction

Vaccines and few anti-viral treatment strategies are currently in use to prevent and manage the clinical symptoms and consequences of severe acute respiratory syndrome coronavirus-2 (SARS-CoV-2, COVID-19) infection. Since virus copies itself resulting in mutations, COVID-19 variants may develop more serious clinical problems. This necessitates the development of new vaccines. Also, there are continuing interest and many efforts in the development of new antiviral drugs and in the repurpose of existing ones, such the HIV medicine, lopinavir and ritonavir. Remdesivir, a broad-spectrum antiviral with characteristics of inhibiting RNA-dependent polymerase, shows great potential among the known antiviral medicines [1]. Even at low micromolar doses, Remdesivir effectively prevents SARS-CoV-2 infection in vitro [2] and in the treatment of COVID-19 patients [3]. Another medicine that has been examined is favipiravir, which was developed in China to treat new influenza [1]. It is a novel inhibitor of RNA-dependent RNA polymerase [4]. Chloroquine phosphate, an antimalarial drug, has been demonstrated to have anti-SARS-CoV-2 characteristics and is effective in treating COVID-19, among the known non-antiviral medicines. It has enhanced lung imaging results and prevented pneumonia from getting worse by blocking viral entrance into endosomes [3,5]. Medications such as cepharanthine, selamectin, and mefloquine hydrochloride may also be useful [6]. Social isolation and quarantine, which do not include the use of pharmaceuticals, have emerged as the primary response tactics for containing the virus in the absence of effective vaccinations or medicines. Individuals exhibiting symptoms of COVID-19 are required to remain in quarantine. During the initial stages of the COVID-19 pandemic, individuals in the US were required to undergo a 14-day self-quarantine period following travel to countries with a CDC Level 3 Travel Health Notice or close contact with an infected person [7].

## 2. Anesthesia Procedures

The principal route of infection for COVID-19 is via infected droplets and aerosols, which exposes anesthesiologists, trainees, and nurse anesthetists during the airway management procedures [8]. To guarantee the well-being of patients and healthcare workers alike, it is imperative that anesthesiologists, intensivists, trainees, certified nurse assistants, certified anesthesiology assistants, and other personnel stay current on the latest methodologies and protocols for managing these patients in the perioperative and intensive care unit (ICU).

In China, 3.5% of healthcare workers contracted COVID-19, and the death rate for this group was approximately 0.3% [9]. Because of their frequent and close contact with sick patients and the high likelihood of breathing in infected patient aerosol during airway manipulations, anesthesiologists have an elevated risk of infection compared to other health care workers [10]. Intubation and invasive ventilation were necessary for around 3.2% of COVID-19 patients, according to current statistics. Members of the anesthesia team may be required to provide care to patients who test positive for COVID-19 in emergency rooms, during transfers from the intensive care unit to the operating room (OR) for extracorporeal membrane oxygenation (ECMO), or when transferred to assist with offsite emergency airway management.

### 2.1 Preoperative Preparations

When a patient with COVID-19 arrives, the anesthesia staff should have the operating room (OR) ready specifically for COVID patient and surgical team, marking them as “infectious surgery” [10]. If a negative pressure setting is available, it is advised to disable the positive pressure setting. A patient with COVID-19 should be transported to the designated operating room after the in-room anesthetic care team notifies the OR charge nurse and other clinicians of the necessary arrangements [10]. Patients who require a preoperative anesthesia evaluation who are positive or suspect for COVID-19 should be evaluated by the principal anesthesiologist [10]. It is always a good practice to check the patient’s temperature, heart rate, and respiration rate in addition to the standard preoperative evaluation. A comprehensive respiratory protective device with a powered air purifier and a positive-pressure medical protective hood if available, disposable work caps, goggles, and a full-face shield, as well as fluid-resistant gowns, gloves, and shoe covers should be worn before any physical contact with patients. The correct way to put on personal protective equipment (PPE) includes the following steps: donning scrubs and a hair cover, washing hands thoroughly, donning the mask, inserting inner gloves, donning the coverall, protecting the eyes (goggles or a face shield), protecting the feet, donning the isolation gown, inserting outer gloves, and finally, doing a fit test [10]. Even though most of the PPE is intended for single use, multi-patient visits involving the same infectious disease diagnosis or procedures with a low risk of contamination may necessitate the usage of gowns during times of urgent shortage [11]. It is possible to wear surgical masks or N95 respirators for long periods of time while caring for many patients. It is suggested that elective treatments requiring PPE be postponed and that the usage of washable PPE be increased [11].

### 2.2 Anesthesia

Anesthesia is advised for the patient so that they do not breathe in any particles or droplets that may be inhaled through their nose and mouth. A spinal anesthetic is still the gold standard for a COVID-19 carrier mother undergoing a cesarean section, and she should always wear a surgical mask or a N95 mask [10]. Because of the potential for aerosolizing airway secretions during bag mask ventilation, which could lead to a higher risk of viral exposure and transmission, rapid sequence induction is advised [12]. When it comes to protecting staff from aerosols and droplets, it is recommended to use a “Negative Pressure Airway Hood” [11] wherever possible. Use the induction and paralysis agents quickly after ensuring enough preoxygenation; patients with severe anxiety may benefit from 1–2 mg of intravenous midazolam for sedation. Suppressing airway reflexes during endotracheal intubation is effectively accomplished with 1.5 mg/kg of intravenous lidocaine. The risk of contagious respiratory infections, such as COVID-19, is higher during tracheal intubation because the patient may spray bodily fluids or blood or create aerosols or droplets [13]. The scarcity of personal protective equipment has led some hospitals to appoint the most seasoned anesthesiologist to work as the main intubator in charge of a particular operating room and shift, outfitted with N95 masks, goggles, and any other necessary gear [14]. It would be best to utilize video laryngoscopy to ensure a safe working distance between the intubator and the airway, and anesthesiology team should double-check that the standard breathing circuit is securely connected before the procedure [14]. Taking a long-acting muscle relaxant before the procedure can help reduce coughing. Inflating the cuff before ventilation, checking the tube position with end tidal CO2, and having a ventilator ready to go right before extubation are all wise decisions [12]. To minimize the transmission of SARS-CoV-2, medical staff caring for patients should change gloves after airway manipulation before using other operating room devices, such as the anesthesia computer or anesthesia cart [11]. After the patient’s expected supplies have been collected, a transparent plastic sheet can be placed over the anesthesia cart to prevent the spread of germs.

### 2.3 Anesthesia Recovery

If a COVID-19 patient needs to be transferred after surgery, it is best to do it in a separate intensive care unit room rather than the post anesthesia care unit. But when the healthcare system becomes overburdened, as it often does in big epicenters like New York, such policies might be upended. If the condition of the patient is stable, extubation can be done in the operating room where it was first performed [13]. Two layers of moist gauze can be placed over the patient’s mouth and nose prior to extubation to reduce the likelihood of intubation site exposure to secretions [10]. In the intensive care unit, tracheal intubation needs to be done promptly if the patient’s condition does not improve after 2 hours of high-flow nasal catheter oxygen therapy or noninvasive ventilation (shown as respiratory distress, breathing frequency >30 times/min, and oxygenation index <150 mm Hg), and if standard oxygen therapy fails to alleviate their respiratory distress and/or hypoxemia [10].

### 2.4 Transporting Patients

Outside of the operating room, it is best for healthcare providers to wear PPE, and a dedicated elevator should be used to transfer patients while they are covered in disposable surgical linen. To prevent unconnected persons from being exposed, one should empty the transfer channel in advance. Regardless of whether the patient is intubated or not, clinicians should always wear medical protective masks during transport. Patients are also required to wear surgical or N95 masks during the transfer [15].

### 2.5 Post Anesthesia Equipment Care and Disposal of PPE

All anesthetic tools, including the video laryngoscope blade and reinforced tubes, should be single-use and disposed of correctly to prevent contamination. Each bag of medical waste should be sealed and either sprayed with chlorinated disinfectant or covered with an extra bag before being removed from the contaminated area. Each bag should be labeled with the following information: “COVID-19,” the name of the department or institute, the date, the time, and the category [10]. Hydrogen peroxide (2–3% concentration), chlorine disinfectant wipes (2–5 g/L concentration), or alcohol wipes (75% concentration) should be used to clean and disinfect any non-disposable anesthetic equipment [10]. It is imperative that all medical staff members participating in the actual surgical procedure remove their personal protective equipment and dispose of it in accordance with the local infection control policy’s specified biohazard disposal bags or containers. Items that cannot be disposed of in a regular trash can should be placed in specific areas for special handling, such as protective apparel. Properly removing personal protective equipment (PPE) should be done in the following order: remove shoe cover, then remove outer gloves, disinfect hands, remove protective clothing, disinfect hands again, remove goggles and protective face screen, remove mask, disinfect hands again, remove hat, disinfect hands again, remove inner gloves, and finally, change into personal clothing [13]. To reduce the possibility of self-contamination, the American Society of Anesthesiologists recommends that providers use extreme caution when removing and disposing of personal protective equipment (PPE). It is recommended to wash hands with soap and water for a minimum of 20 seconds or use an alcohol-based hand rub containing 60% to 95% alcohol for proper hand hygiene [11].

### 2.6 Management of Medical Staff upon Exposure to COVID-19

Health care workers should promptly notify their supervisor or occupational health services after an exposure and evaluate their risk of COVID-19 exposure in accordance with the US CDC standards [16]. Highly susceptible healthcare personnel should refrain from interacting with patients for 14 days following their last exposure to a confirmed COVID-19 patient, undergo a COVID-19 test, and be isolated for the same amount of time [17]. Daily throughout the observation period, the patient’s temperature and any signs of respiratory distress are recorded. But, after consulting with occupational health services, it is permissible for a health care worker who has been exposed but shows no symptoms to work if there is a significant shortage of medical staff and all other options for enhancing staffing have been tested and failed [18]. It is imperative that they consistently wear facemasks while on the clock, refrain from coming into touch with patients who have highly impaired immune systems, and practice good respiratory and hand hygiene [18]. For 14 days following their last contact with a COVID-19 patient, low-risk healthcare workers should self-monitor their temperature and respiratory symptoms. If they develop any symptoms suggestive of COVID-19, they should notify their healthcare facility immediately. As a reminder, health care workers should reinforce droplet and contact precautions [18]. Due to the lack of certainty regarding whether patients who have recovered with COVID-19 can potentially transmit SARS-CoV-2, follow-ups are necessary in additional medical procedures involving patients who have not tested positive for the virus.

## 3. COVID-19 Infection in Obstetrics

### 3.1 Clinical Manifestation of Covid-19 Infection in Pregnancy

In a study of Chinese pregnant patients with COVID-19, the symptoms reported were as follows: fever (99%), exhaustion (70%), cough (59%), difficulty breathing (31%), myalgias (35%), headache (6.5%), sore throat (17%), diarrhea (10%), nausea (10%), and vomiting (4%). As part of the screening process for COVID-19 infection, the American Academy of Otolaryngology-Head and Neck Surgery presently recommends looking for symptoms such as a rapid loss of smell or taste [20].

Although the symptoms of COVID-19 infection during pregnancy are like those of other common pregnancy and labor conditions, many of these nonspecific symptoms may be caused by the virus itself [21]. For instance, clinicians may fail to recognize COVID-19 infection because symptoms such as myalgias and diarrhea are indicators of latent labor, headaches are symptoms of preeclampsia, and tachycardia and fever are symptoms of chorioamnionitis, respectively, during pregnancy and labor. Furthermore, COVID-19 infection in pregnant women might go unnoticed until the baby is born or even later in life [22], which creates a serious risk of infection for the mother, her family, and any healthcare professionals who treat the patient.

It is recommended to isolate women who test positive for COVID-19 or have a high risk of contracting the virus during pregnancy. Whenever aerosolizing operations are to be performed, it is imperative to utilize airborne infection isolation rooms, which are single-patient negative-pressure rooms with a minimum of 6 air changes per hour, if they are available. Typically, it is advised to use isolation rooms that are appropriate for droplet and contact precautions [23]. The same guidelines that apply to patients in general should be followed when planning transportation, strategies for reducing exposure, and cohorting for patients who are under investigation or already diagnosed with COVID-19 infection [17].

### 3.2 Patients under Pregnancy Labor with Active Covid-19 Infection

The use of neuroaxial analgesia during pregnancy labor is still recommended by obstetricians, even when COVID-19 is present. If an intrapartum cesarean section is necessary, it is preferable to insert an epidural early so that respiratory problems during labor do not worsen and thus to avoid the use of general anesthesia [23].

By reducing the risks of aerosol exposure and the transmission of COVID-19 infection during intubation and extubation, neuraxial analgesia is beneficial in the setting of COVID-19 pneumonia for both the patient and the healthcare providers. For the patient, it helps avoid any worsening of respiratory status during intubation and mechanical ventilation [23].

The anesthesiologist administering the neuraxial labor analgesia should not be at high risk of contracting COVID-19 because the method does not produce aerosols. To avoid cross-contamination, everyone providing patient care should wear protective gear, including an impermeable gown, gloves, and a surgical mask and goggles. No more than two people should be present when inserting neuraxial labor analgesia, and the patient should always wear a surgical mask to prevent the spread of droplets [23].

In the context of COVID-19, “there is insufficient information about the cleaning, filtering, and potential aerosolization of nitrous oxide.” This is according to the current guidelines for the use of nitrous oxide as a labor analgesic. It may be prudent for individual labor and delivery units to cease use. The use of high-flow oxygen to alleviate fetal distress is also associated with an increased risk of aerosolization and has no beneficial effect on fetal outcomes.

### 3.3 Anesthesia for Cesarean Delivery

Many pregnant women in China who tested positive for COVID-19 had caesarean births [24]. The presence or absence of COVID-19 infection may not be identified prior to a caesarean section if testing is not universally available, and findings are not processed quickly. One study found that 5% of attempts to switch from labor epidural analgesia to caesarean delivery anesthesia would fail [25]. If you’re considering converting from intrapartum neuraxial labor analgesia to cesarean delivery anesthesia, it’s important to keep in close contact with your obstetricians. This will ensure a safe transfer to the operating room and give you enough time to start the surgical block, so you don’t have to go under general anesthesia [25]. Unless the patient is officially determined to be COVID-19 negative, all personnel in the operating room should wear airborne protection, specifically a N95 respirator mask, to reduce the risk of exposure during urgent endotracheal intubation.

In a study conducted in Wuhan, China, researchers examined the effects of epidural anesthesia on blood pressure levels during cesarean deliveries. They found that out of 17 women who tested positive for COVID-19, 14 had “excessive hypotension” compared to 3 who had received general anesthesia. Unfortunately, the study did not report any data regarding the trends in blood pressure or the use of vasopressors [26]. Spinal anesthetic was well-tolerated and resulted in stable blood pressure in a larger case series of 49 patients, including 45 who underwent cesarean deliveries and 4 who underwent orthopedic surgeries [27].

### 3.4 Cardiac Anesthesia during the COVID-19

The cardiac anesthesia team faces several obstacles when patients with suspected or confirmed COVID-19 infection undergo cardiac surgical operations. They may indicate increased risks of perioperative morbidity and death and call for a more cautious approach during perioperative anesthetic management. The perioperative context requires extra caution due to the high stakes involved in managing COVID-19 infected cardiovascular patients and ensuring the safety of all professionals involved [28].

While cardiac surgery and associated anesthesia may not be directly involved in the treatment of COVID-19 patients, the expanding coronavirus pandemic has had a significant influence on this area of surgery and anesthesia. There are a lot of ways the pandemic has already impacted cardiac surgery units. For example, there aren’t enough intensive care unit beds and ventilation sites, elective and complex cardiac interventional procedures have to be delayed or canceled, patients get COVID-19 after cardiac surgery, patients with coronavirus need urgent cardiac operations, cardiac anesthesiologists have to transfer within the hospital to staff and support the ICUs in front of the pandemic, there aren’t enough medical and nursing practitioners because of the infection, clinical meetings are limited, and CME and training have been cancelled [29].

### 3.5 Anesthetic Preoperative Preparation and Management of Suspected or Confirmed COVID–19 Patients

First, the heart surgery operating room requires a pair of seasoned cardiac anesthesiologists and a cardiac anesthesia nurse to oversee the anesthetic treatment of patients, both for the sake of efficient staffing management and because of the higher risk involved with cardiac procedures. Preparation is key, thus have a third cardiac anesthesiologist on call outside of the operating room to provide backup and advice [30].

Patients undergoing cardiac surgery are required to always wear a N95/surgical mask and are to be carried to the operating room using a certain planned route. In cases where it is necessary, nasal oxygen therapy can be administered under the surgical mask. In general, you should stay away from a Venturi mask [13]. Extracorporeal membrane oxygenation (ECMO) or intracardiac bypass grafting may be considered in patients with considerable pulmonary and cardiac dysfunction [31].

### 3.6 Intraoperative Management of Suspected or Confirmed COVID–19 Cardiac Surgical Patients

The use of ultrasound guiding during arterial and cardiovascular catheterization has several benefits, including increased success rates, shorter procedure times, and the prevention of blood contamination of nearby staff due to repeated vascular punctures [32]. When possible, surgeons would rather use regional anesthesia than general anesthesia for surgical procedures. When it comes to heart surgery, though, a single regional anesthesia approach would not be adequate. Depending on the procedure, it can be used in conjunction with general anesthesia to help alleviate perioperative pain [33]. Quick sequence induction and endotracheal intubation are the gold standards, with succinylcholine or rocuronium—two fast-acting muscle relaxants—being the go-to solutions in case of an emergency. To prevent deadly situations of severe desaturation, patients with inadequate oxygen reservoirs might be administered 100% oxygen through a low-pressure mask breathing system [34]. The use of endotracheal intubation and rapid sequence induction are both highly suggested. As a first line of defense, quick-acting muscle relaxants like succinylcholine or rocuronium could be used. To prevent serious desaturation situations, patients with weak oxygen reserve might be given low-pressure mask ventilation with 100% oxygen [34].

### 3.7 Postoperative Processes for Cardiac Surgery Patients Suspected or Confirmed to Have COVID-19

An interdisciplinary cardiac COVID-19 team is required to provide postoperative care and rigorous follow-up for COVID-19 patients. This team should primarily consist of anesthesiologists, cardiovascular surgeons, respiratory medicine doctors, infectious diseases experts, seasoned nurses, physiotherapists, social workers, and other trained professionals. [35].

## 4. COVID-19 Implications for Pediatric Anesthesia

Childhood instances of COVID-19 account for 1% to 5% of all cases, according to data from China, the US, and Italy [36]. By April 14, 2020, 186 ICU COVID-19-related hospitalizations and three deaths had been recorded by the Virtual Pediatric Systems Collaborative Group, which collects data from pediatric intensive care units at 177 institutions, the majority of which are in the United States [37].

One thing that makes perioperative care different for pediatric patients is that parents are usually present in the operating room. Many pediatric centers have made it standard practice to let one parent accompany their child to the operating room or procedure room and stay through the inhalational induction of general anesthesia, even though this practice is controversial regarding whether it reduces perioperative anxiety in children and parents [37].

While following the advice, there are a few things to think about. During the induction of anesthesia, children have a lower tolerance for apnea. Even while classical quick sequence intubation effectively decreases aerosol production, it may be required to ventilate with a low pressure (10--12 cm H_2_O) face mask until the medicines take effect, especially for smaller children [38]. If intubation fails, the laryngeal mask can be quickly and effectively inserted as an alternative. If minimal pressures are utilized and no air leakages are seen, it should be considered for patients with no symptoms or low suspicion of COVID-19.

It is important to consider other pertinent matters in pediatric anesthesia in this specific situation. To minimize the chances of infection, the operating room staff should consist of no more than what is necessary for a safe induction of anesthesia, such as two anesthetists and one anesthesia assistant. Given the circumstances, it is not recommended that parents be present [39]. Subsequently, preanesthetic medicine becomes increasingly important for easing preoperative anxiety and making vein puncture easier. Medications like midazolam and ketamine, administered orally or intramuscularly, are generally considered safe alternatives. Despite their growing usage in pediatric anesthesia, alfa-2 adrenergic agonists aren’t without their drawbacks, such as a sluggish onset of action and potential difficulties with intranasal delivery. The use of venous induction is advised, as mentioned earlier [39]. While inducing inhalation, exercise caution to avoid positive pressure ventilation through the face mask if venous access cannot be obtained in advance. Some questions will be answered and many more will be raised as we gain more knowledge about the behavior of the new SARS-CoV-2 virus.

There is an immediate need to gather more high-quality evidence for the pediatric population; improve the description of occupational exposure risk for health professionals like anesthetists; and quickly ascertain the outcomes of the actions and interventions implemented in response to this situation. Until then, for an unknown amount of time, children will be sedated in a less “pediatric” way according to protocols designed for adults [40].

## 5. Effects of Anesthetics on Immune System Modulation in Critically Sick Patients with COVID-19

Maintaining a steady state of the inflammatory response is crucial for the probability of survival of COVID-19 patients. There is a strong cytokine storm in COVID-19 infection [41]. Also, COVID-19 patients may develop cardiovascular disorders [42, 43], hematological complications [44–46], and clinical conditions. Indeed, gut-brain communication in COVID-19 has been reported and the findings provide some information for the diagnostic biomarkers and therapeutics [47]. Interestingly, ocular transmission mode of virus modes of virus has also been reported [48]. Accordingly, the healthcare providers, including anesthetics can have both pro- and anti-inflammatory effects on the immune system, depending on the individual.

Endotracheal intubation and mechanical ventilation should be initiated promptly for critically sick individuals with COVID-19 who also have acute hypoxemic respiratory insufficiency or failure. The recommended strategy for inducing anesthesia is rapid-sequence induction (RSI), and it may be necessary to administer anesthetic drugs cautiously. Some medications are suggested for RSI in COVID-19 patients, but otherwise the fundamentals of airway care are the same as in more controlled environments [12].

### 5.1 Immunomodulatory Properties of Intravenous Anesthetic Agents

The impact of anesthetics on immune-system modulation may vary and includes both pro- and anti-inflammatory effects [49]. The immune status of the patient is the most important factor to consider when choosing and using anesthetics for COVID-19. This is particularly true for obese patients, as the inflammatory condition is worsened by the combined effects of inflammation-induced changes in tissue-resident macrophages to an M1-like phenotype and the expression of cytokines and adipokines by adipocytes [50].

Anesthetics also significantly improve inflammation resolution, which is a major benefit. By inhibiting the secretion of pro-inflammatory cytokines and chemokines and stimulating the clearance of cellular debris, endogenous pro-resolving bioactive mediators can halt the inflammatory response [51]. In viral disorders, a breakdown of mechanisms that normally resolve inflammation keeps pathologic inflammation going [52], which suggests that it could play a role in maintaining dysregulated inflammation and the deaths that come with it in COVID-19 [53]. Epoxyeicosatrienoic acids (EETs), which are generated from arachidonic acid, also have a role in promoting inflammation resolution. A few important pro-inflammatory cytokines can be inhibited by these mediators, which also regulate gene expression and ion transport, relax blood vessels, aid in the removal of cell debris, and activate anti-inflammatory pathways [52].

These mechanisms can be altered by different anesthetics. It is well-established that 5-lipoxygenase produces leukotrienes as a byproduct of arachidonic acid metabolism [54]. Among other potential effects, propofol may reduce leukotriene synthesis in DCs and other immunological and nonimmune cell types by inhibiting 5-lipoxygenase [55]. Additionally, dexmedetomidine has been demonstrated to increase levels of the inflammation-resolving orchestrator netrin-1, which in turn increases levels of the pro-resolving humoral mediator lipoxin and decreases levels of the pro-inflammatory humoral mediator leukotriene B4 [56]. Enhanced EET synthesis from arachidonic acid by cytochrome P-450 epoxygenases is associated with a metabolic shift toward inflammation resolution [57]. For individuals suffering from low-grade or chronic inflammation, who should prioritize reducing the severity of their disease, it is crucial to improve the generation of EETs from arachidonic acid by cytochrome P-450 epoxygenases. Inducing a decrease in cyclooxygenase enzyme activity, as propofol does, can further improve this mitigation [58]. Nevertheless, soluble epoxide hydrolase quickly converts ETTs, which constrict blood vessels in the lungs, into dihydroxyeicosatrienoic acids, which have less of an effect on blood vessel dilation [59]. Thus, propofol may work better when given early in the inflammatory process; however, in cases of severe respiratory failure accompanied by a cytokine storm, it may be more effective to combine propofol with a pulmonary vasodilator (such as intravenous epoprostenol or inhalational iloprost) and a soluble epoxide hydrolase inhibitor (such as urea, carbamate, or amide derivatives) [60]. Nevertheless, these combinations necessitate more evaluation in rigorous studies.

### 5.2 Potent Effects of Etomidate in Patients with COVID-19

Emergency anesthesia often makes use of etomidate, a short-acting intravenous anesthetic medication that has long been the drug of choice for RSI and endotracheal intubation [61].

The use of etomidate in cases of septic shock was questioned in 2008 due to the results of CORTICUS (Hydrocortisone Therapy for Patients with Septic Shock) [62], a multicenter, randomized, double-blind, placebo-controlled trial. The trial suggested that etomidate may either reduce the likelihood of shock reversal or prolong its reversal. According to another study, individuals with septic shock who were treated with etomidate were more likely to be nonresponsive to corticotropin, and administering hydrocortisone had no impact on their outcome [63].

A 1983 investigation in trauma victims presented the first evidence of the connection of etomidate with “late death” [64]. Retrospective research conducted 20 years after etomidate was first prescribed found that the drug raised the likelihood of inflammatory organ injury, with patients exhibiting adrenal suppression, ARDS, or MODS at a higher risk in those with an APACHE score greater than 20 [65].

This disturbing data may have crucial implications for the treatment of COVID-19 patients in critical care, where maintaining a stable inflammatory response seems to be a key factor in determining prognosis. Etomidate may also increase the risk of secondary infections and corticosteroid dysfunction [66], as well as lengthen the systemic inflammatory response syndrome in COVID-19 patients [67] by increasing pro-inflammatory cytokine production ex vivo in whole-blood cell cultures challenged with lipopolysaccharide. As an alternative, etomidate’s effect on cortisol levels may dampen DC homing, monocyte and neutrophil activity, and other immune system processes [68]. Also, inflammation-related problems like ARDS and MODS are more likely to occur when cortisol levels are low because this hormone hinders the preservation of endothelial integrity and vascular tone, increases phospholipase A2 synthesis, and hinders leukocyte death [69].

## 6. Conclusion

Ropivacaine is a safe alternative to bupivacaine as a long-acting local anaesthetic for supraclavicular brachial plexus block. The study suggests that ropivacaine provides a quicker onset of both sensory and motor block, a longer duration of anaesthesia and analgesia, that offers a comparable quality of block to bupivacaine. Moreover, ropivacaine has the added benefit of extending the time before the first rescue analgesic is needed in the postoperative period, compared to bupivacaine.

## Figures and Tables

**Figure 1:**
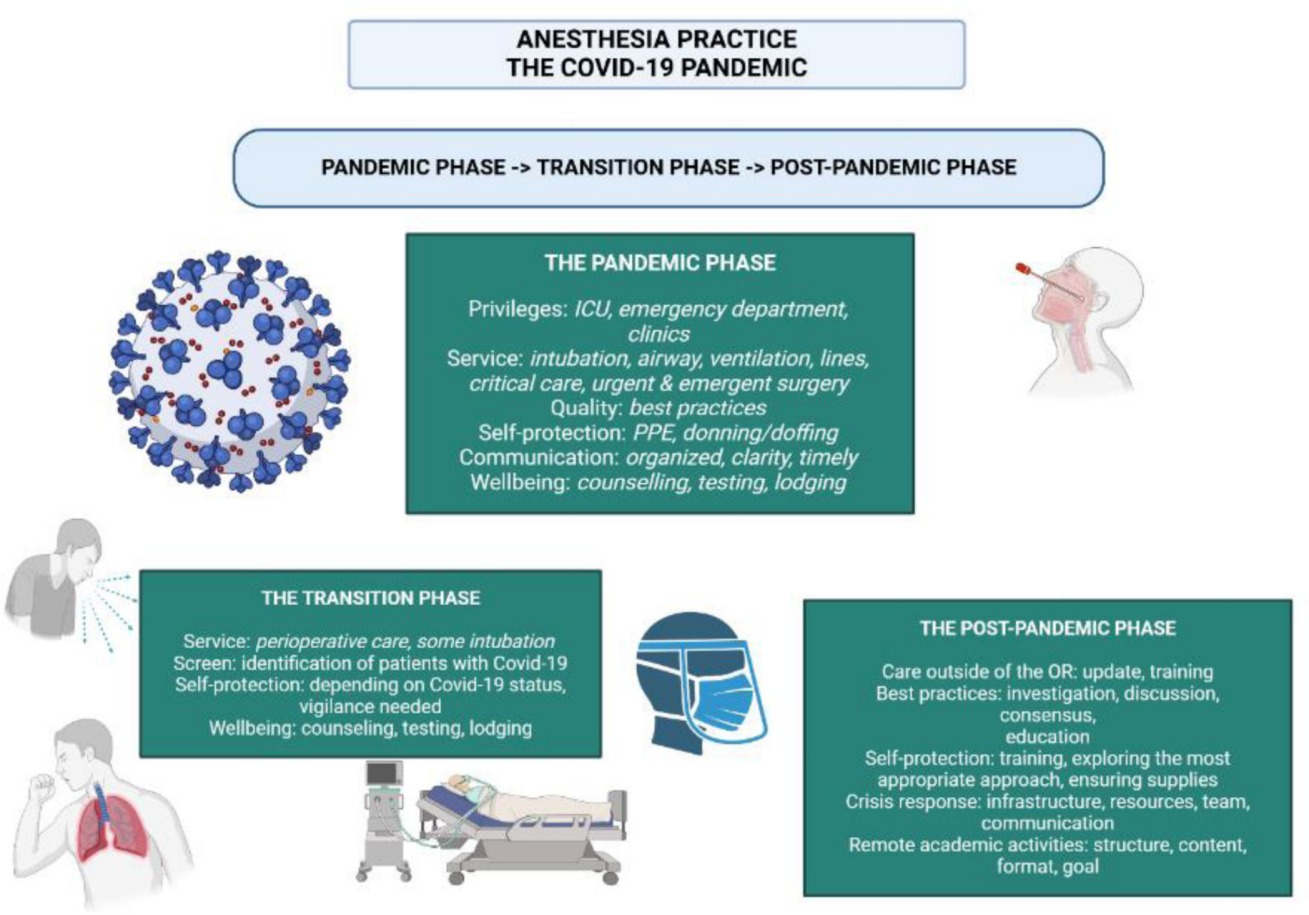
Impact of the Covid-19 pandemic on anesthesia practice through different phases.

**Figure 2:**
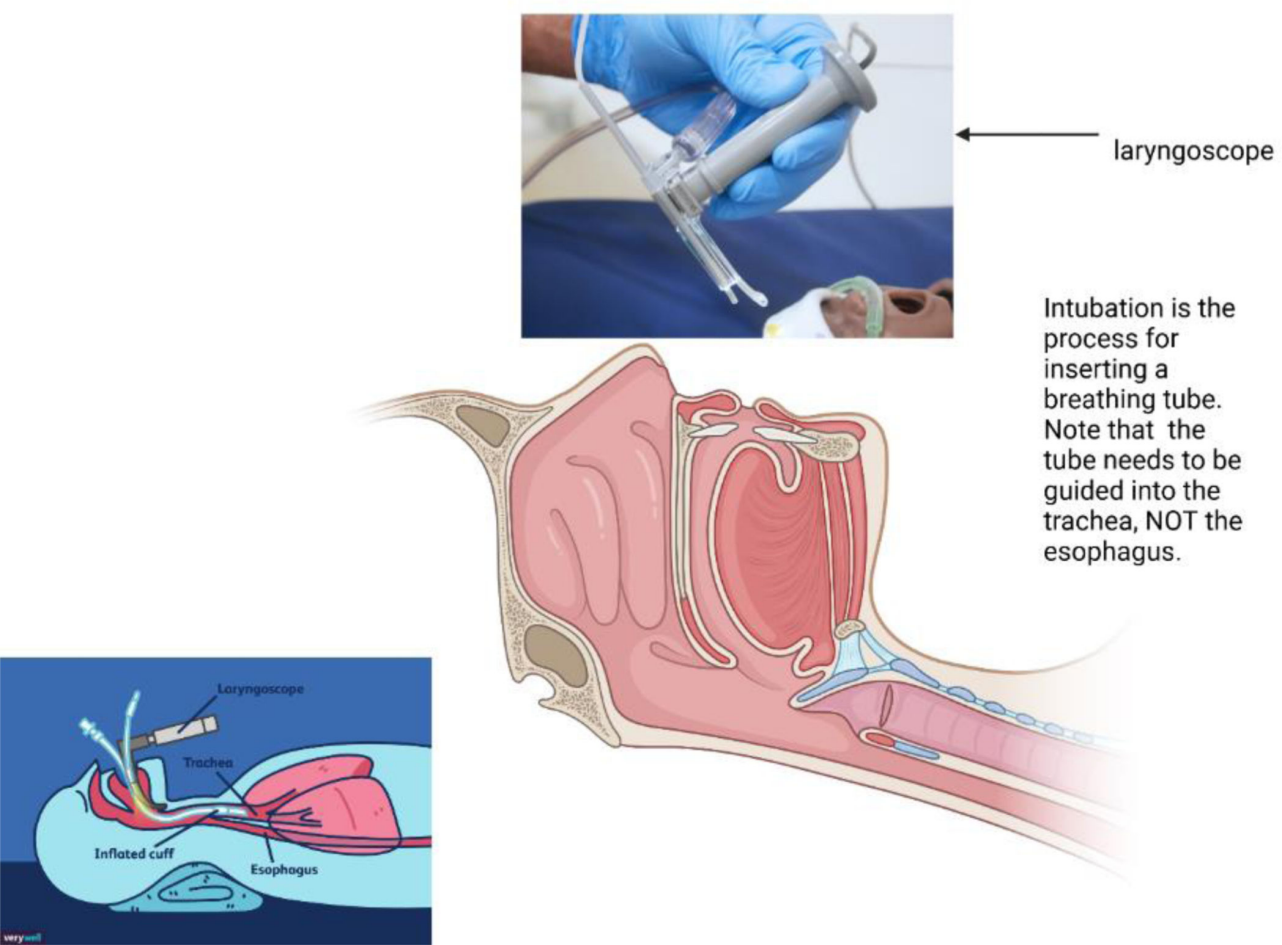
Schematic diagrams showing Rapid Sequence Intubation (RSI).

**Figure 3:**
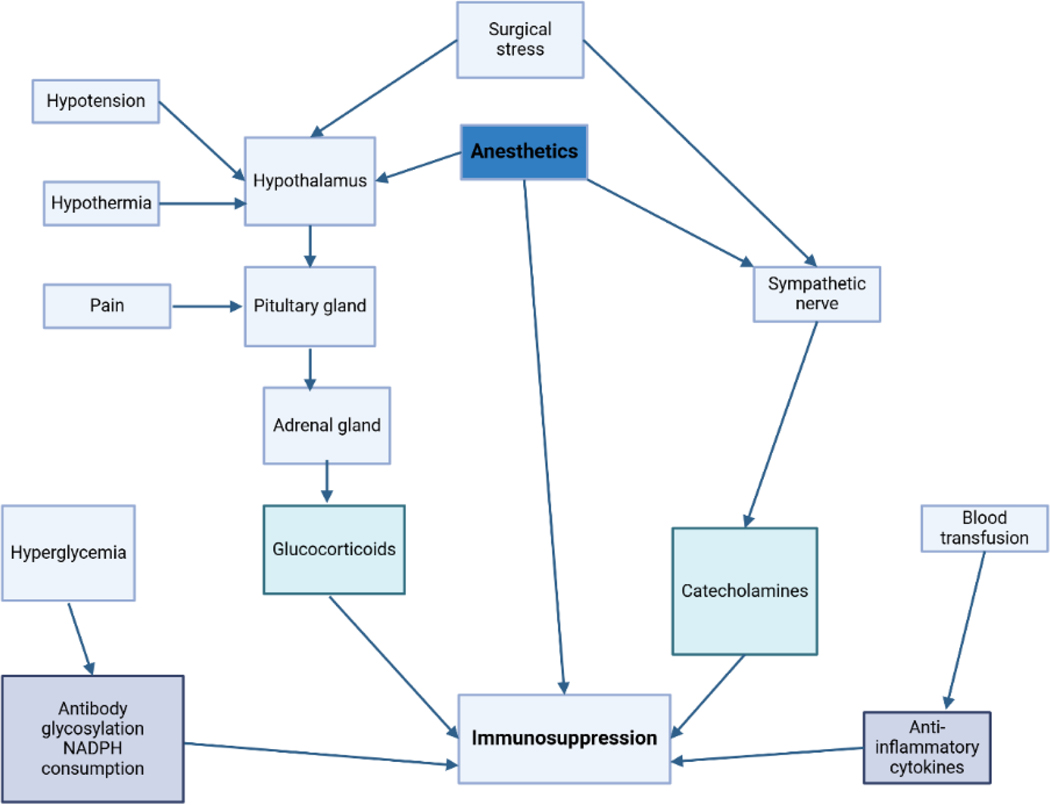
Potential anesthetic-induced immunological competency modulators depicted schematically. While under anesthesia, the immune system is directly and indirectly impacted by neuro-immune-endocrine interactions that occur.

**Figure 4:**
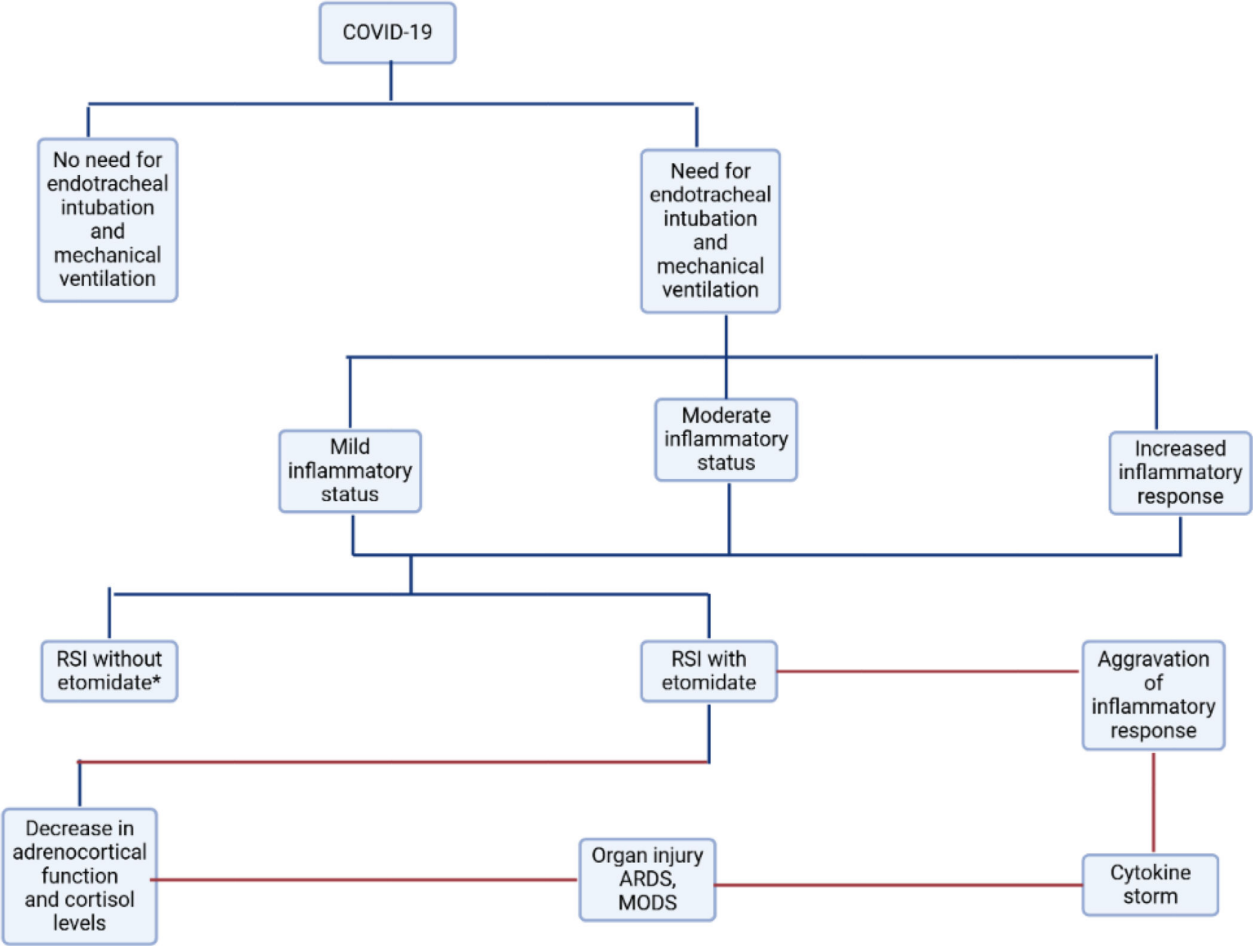
Patients with COVID-19 who require endotracheal intubation or mechanical ventilation will be managed according to the steps shown in the graphic. It lays out the procedure and its dangers for patients with mild to moderate inflammatory states, including RSI with or without etomidate, and the possible outcomes, such as reduced adrenocortical function, organ damage (ARDS, MODS), and cytokine storm.

## Key Points

- Anesthesia staff are at higher risk of COVID-19 exposure during intubation and airway management procedures, which produce aerosols.
- PPE protocols are critical for reducing the risk of viral transmission in operating rooms, especially during procedures like intubation.
- The use of regional anesthesia reduces the need for airway manipulation, lowering exposure risk during surgical procedures.
- Remdesivir, favipiravir, and chloroquine are among the antiviral drugs explored for treating COVID-19, with remdesivir showing some beneficial effect.
- Obstetric anesthesia during COVID-19 focuses on neuraxial anesthesia to minimize aerosol generation and improve outcomes for both patients and staff.
- In cardiac anesthesia, the COVID-19 pandemic has led to delays or cancellations of elective surgeries and increased risks during perioperative care.
- Special considerations are made for pediatric anesthesia, as children have lower tolerance for apnea, necessitating cautious airway management.
- Immunomodulatory effects of anesthetics play a role in critically ill COVID-19 patients, with some agents like propofol having both pro- and anti-inflammatory effects.
- Post-surgical management for COVID-19 patients involves stringent follow-up and care, especially in cases with respiratory complications.
